# The Influence of the Auxiliary Ligand in Monofunctional Pt(II) Anticancer Complexes on the DNA Backbone

**DOI:** 10.3390/ijms25126526

**Published:** 2024-06-13

**Authors:** Evanthia-Vasiliki Tagari, Evangelia Sifnaiou, Theodoros Tsolis, Achilleas Garoufis

**Affiliations:** 1 Laboratory of Inorganic Chemistry, Department of Chemistry, University of Ioannina, 45110 Ioannina, Greece; ch05512@uoi.gr (E.-V.T.); e.sifnaiou@uoi.gr (E.S.); t.tsolis@uoi.gr (T.T.); 2 Institute of Materials Science and Computing, University Research Centre of Ioannina (URCI), 45110 Ioannina, Greece

**Keywords:** monofunctional Pt(II) complexes, guanosine, sugar conformation, 9-MeG, hydrolysis rate

## Abstract

Monofunctional platinum complexes offer a promising alternative to cisplatin in cancer chemotherapy, showing a unique mechanism of action. Their ability to induce minor helix distortions effectively inhibits DNA transcription. In our study, we synthesized and characterized three monofunctional Pt(II) complexes with the general formula [Pt(en)(L)Cl]NO_3_, where en = ethylenediamine, and L = pyridine (py), 2-methylpyridine (2-mepy), and 2-phenylpyridine (2-phpy). The hydrolysis rates of [Pt(en)(py)Cl]NO_3_ (**1**) and [Pt(en)(2-mepy)Cl]NO_3_ (**2**) decrease with the bulkiness of the auxiliary ligand with k(**_1_**) = 2.28 ± 0.15 × 10^−4^ s^−1^ and k(**_2_**) = 8.69 ± 0.98 × 10^−5^ s^−1^ at 298 K. The complex [Pt(en)(2-phpy)Cl]Cl (**3**) demonstrated distinct behavior. Upon hydrolysis, an equilibrium (K_eq_ = 0.385 mM) between the complexes [Pt(en)(2-phpy)Cl]^+^ and [Pt(en)(2-phpy-H^+^)]^+^ was observed with no evidence (NMR or HR-ESI-MS) for the presence of the aquated complex [Pt(en)(2-phpy)(H_2_O)]^2+^. Despite the kinetic similarities between phenanthriplatin and (**2**), complexes (**1**) and (**2**) exhibit minimal activity against A549 lung cancer cell line (IC_50_ > 100 μΜ), whereas complex (**3**) exhibits notable cytotoxicity (IC_50_ = 41.11 ± 2.1 μΜ). In examining the DNA binding of (**1**) and (**2**) to the DNA model guanosine (guo), we validated their binding through guoN7, which led to an increased population of the C3′-*endo* sugar conformation, as expected. However, we observed that the rapid transition ^2^E (C2′*-endo*) ↔ ^3^E (C3′*-endo*), in the case of [Pt(en)(py)(guo)](NO_3_)_2_ ([**1**-guo]), slows down in the case of [Pt(en)(2-mepy)(guo)](NO_3_)_2_ ([**2**-guo]), resulting in separate signals for the two conformers in the ^1^H NMR spectra. This phenomenon arises from the steric hindrance between the methyl group of pyridine and the sugar moiety of guanosine. Notably, this hindrance is absent in [**2**-(9-MeG)] (9-MeG = 9-methylguanine), probably due to the absence of a bulky sugar unit in 9-MeG. In the case of (**3**), where the bulkiness of the substitution on the pyridine is further increased by a phenyl group, we observed a notable proximity between 9-MeGH8 and the phenyl ring of 2-phpy. Considering that only (**3**) exhibited good cytotoxicity against the A549 cancer cell line, it is suggested that auxiliary ligands, L, with an extended aromatic system and proper orientation in complexes of the type cis-[Pt(en)(L)Cl]NO_3_, may enhance the cytotoxic activity of such complexes.

## 1. Introduction

Platinum-based drugs are a cornerstone of cancer chemotherapy. Since the discovery of cisplatin in the 1960s and its notable efficacy against various tumor types, other drugs, such as carboplatin and oxaliplatin, have been discovered and have gained FDA approval for global clinical use. These compounds are structural analogues of cisplatin, sharing a similar bifunctional motif of action by forming inter- and intra-strand crosslinks with DNA, inducing damage and eventually leading to cell apoptosis [1]. However, they often exhibit significant side effects, including nephrotoxicity, neurotoxicity, ototoxicity, and myelosuppression [2]. The lack of specificity in targeting cancer cells, along with reduced effectiveness due to resistance in specific cancer types [1,3], highlights the necessity for prioritizing research on novel compounds with unique mechanisms of action.

In addressing these challenges, the deployment of cationic monofunctional platinum (II) complexes emerges as an innovative approach in cancer chemotherapy [4,5]. Unlike the simple monofunctional complexes like [Pt(NH_3_)_3_Cl]^+^ and [Pt(dien)Cl]^+^, which have shown inactivity against cancer cells [6,7,8], a pioneering study by Hollis et al. in the late 1980s revealed that a specific class of monofunctional complexes, known as *cis*-[Pt(NH_3_)_2_(L)Cl]^+^ (where L is a nitrogen-donor ligand), exhibited significant inhibitory effects on tumor cell growth, both in vitro and in vivo, particularly against leukemia L1210 and P388 [9]. However, the proposed mechanism of action emerged thirty years later [10], elucidated through the study of the *cis*-[Pt(NH_3_)_2_(pyridine)Cl]^+^, also known as pyriplatin. X-ray crystallography data reveal that RNA polymerase II (pol II) stalled at the pyriplatin adduct on a DNA strand, suggesting that these monofunctional DNA adducts hinder transcription via a mechanism distinct from that of cisplatin. This hindrance is attributed to the pyridine auxiliary-ligand, which explains why pyriplatin inhibits transcription, whereas adducts formed by other monofunctional platinum compounds lacking bulky groups, such as [Pt(dien)Cl]Cl and [Pt(NH_3_)_3_Cl]Cl, exhibit significantly lower inhibitory potency [11].

To enhance the anticancer efficacy of monofunctional platinum complexes, while also considering structural requirements, a series of compounds with various N-heterocyclic (Am) ligands of the formula *cis*-[Pt(NH_3_)_2_(Am)Cl]^+^ was synthesized [12]. Among them, *cis*-[Pt(NH_3_)_2_(phenanthridine)Cl]^+^ emerged as the most potent cytotoxic agent, demonstrating IC_50_ values 7–40 times lower than those of cisplatin or oxaliplatin across a range of human cancer cell lines. The steric hindrance of the bulky and hydrophobic phenanthridine, which is perpendicular to the platinum coordination plane, enhances cellular uptake and slows down the axial attack of water molecules, contributing to the cytotoxic efficiency of the complex. The slow hydrolysis kinetics of phenanthriplatin offer relative inertness towards cytoplasmic nucleophiles, thereby preventing cellular deactivation. Phenanthriplatin’s DNA action mechanism is similar to that of [Pt(terpy)Cl]^+^, involving rapid partial intercalation of the phenanthridine moiety followed by slower coordination binding to the guanine residues of the DNA [13,14,15,16,17]. Compared to cisplatin, phenanthriplatin causes distinct gene modulation in A549 cell lung cancer, potentially offering clinical advantages in cancers with specific genetic profiles [18].

Another class of monofunctional platinum(II) complexes includes [Pt(en)Cl(AcH^+^)]^+^, were Ac = acridine and its derivatives. Despite their efficacy in inhibiting RNA polymerase II and DNA synthesis in vitro, these complexes have not yielded successful in vivo results [19,20,21]. Finally, considering phenanthriplatin’s role as a Top2 poison [22], several studies have been conducted on novel monofunctional platinum(II) complexes. These efforts aim to target not only critical enzymes involved in tumor progression [23] but also specific organelles such as mitochondria [24] and lysosomes [25] enhancing the anticancer efficacy of these complexes, particularly against cisplatin-resistant cancer cell lines [26].

**Figure 1:**
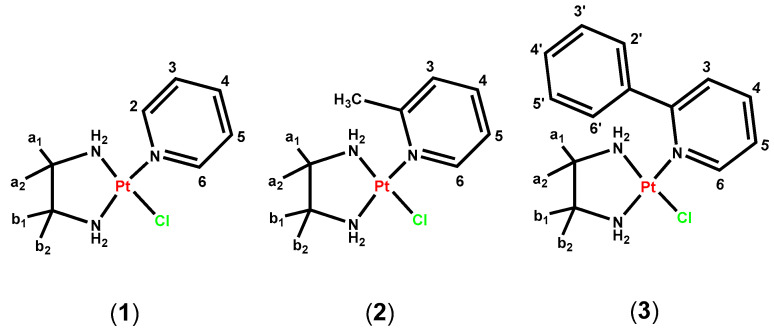
Structures of the studied complexes, with proton numbering.

Taking the above considerations into account, we synthesized and characterized three distinct monofunctional Pt(II) complexes with the general formula [Pt(en)(L)Cl]NO_3_, where en = ethylenediamine and L = pyridine (**1**), 2-methylpyridine (**2**), and 2-phenylpyridine (**3**) (Figure 1). Although complex (**1**) was originally synthesized as its chloride salt by Hollis et al. [9], we chose to synthesize its nitrate salt to examine the hydrolysis of the Pt-Cl bond without being inhibited by the common counterion. This choice also facilitates comparison with the other complexes, (**2**) and (**3**). Additionally, we conducted a detailed characterization using NMR and HR-ESI MS techniques. The primary objectives of this study are: (i) to explore how substituents in the 2-position of a pyridinic auxiliary ligand (L) in [Pt(en)(L)Cl]^+^ affect the hydrolysis kinetics and cytotoxic activity against the A549 cancer cell line, and (ii) to analyze the structural alterations induced by the coordination of these complexes to guanosine and 9-MeG, serving as DNA models.

## 2. Results and Discussion

### 2.1. Synthesis and Characterization of the Complexes (***1***)–(***3***)

**Scheme 1:**
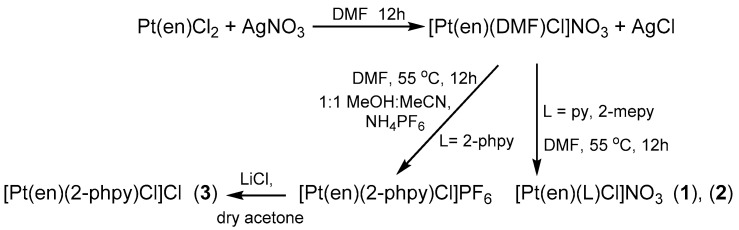
Synthetic procedure and reaction conditions for the complexes (**1**)–(**3**).

The complexes (**1**) and (**2**) were synthesized from the complex Pt(en)Cl_2_ by replacing one [Cl]^−^ with one equivalent AgNO_3_ in DMF, followed by the addition of ligand L, as it is illustrated in Scheme 1. However, (**3**) was synthesized through the precipitated [PF_6_]^−^ salt, which was subsequently converted to [Cl]^−^ in order to purify it from byproducts. The [NO_3_]^−^ salt of (**3**) was formed using AgNO_3_, as explained in the experimental section.

In the HR-ESI mass spectra of complexes (**1**) and (**2**), single-charged cations assignable to [Pt(en)(py)Cl]^+^ and [Pt(en)(2-mepy)Cl]^+^ were observed at *m*/*z* = 369.0430 (**1**) and 383.0604 (**2**), respectively. Additionally, distinct cluster peaks were observed at *m*/*z* = 333.0680 (**1**) and 347.0833 (**2**), attributed to hydrolyzed complexes {[Pt(en)(py)]-H}^+^ and {[Pt(en)(2-mepy)]-H}^+^ (Figures S1 and S2). In the case of complex (**3**), a cluster peak at *m*/*z* = 445.0753 was attributed to the cation [Pt(en)(2-phpy)Cl]^+^, while a single-charged peak at *m*/*z* = 409.0987 was assigned to the cation {[Pt(en)(2-phpy)]-H}^+^ (Figure S3).

In the ^1^H NMR spectra of complexes (**1**)–(**3**), signals from aromatic protons, as well as the ethylenediamine Ha1a2 and Hb1b2-CH_2_-groups, were observed. In the spectrum of complex (**1**), the protons H2/6 shifted significantly downfield by 0.56 ppm compared to the free pyridine, suggesting the coordination of pyridine to the platinum(II) center through its neighboring nitrogen atom (Figure S4). A comparable shift was also reported for the corresponding protons of nicotinic acid in a cyclometalate complex of platinum [27]. Similar downfield shifts were observed in the cases of (**2**) and (**3**) for H6 and H2/6, respectively, with the neighboring methyl group in (**2**) shifting downfield by 0.33 ppm (Figure S5). The chemical shifts of ethylenediamine Ha1a2 and Hb1b2 appear to be affected by the non-symmetric Pt(II) coordination sphere, making them non-equivalent. This phenomenon is consistent with observations in the complexes [Pt(en)Cl(D_2_O)]^+^ and [Pt(en)Cl(OD)], where the ethylenediamine’s -CH_2_- protons were assigned at 2.49 and 2.62 ppm, and 2.45 and 2.61 ppm, respectively [28]. However, in the case of complex (**3**), the intermediate kinetics of the rotation of the Pt-N bond of 2-phpy resulted in the broadening of these signals (Figure S6). This indicates that the phenyl ring of 2-phpy is positioned in such a way that restricts the rapid twist of the ethylenediamine five-membered ring.

### 2.2. Hydrolysis of the Complexes (***1***)–(***3***)

**Figure 2:**
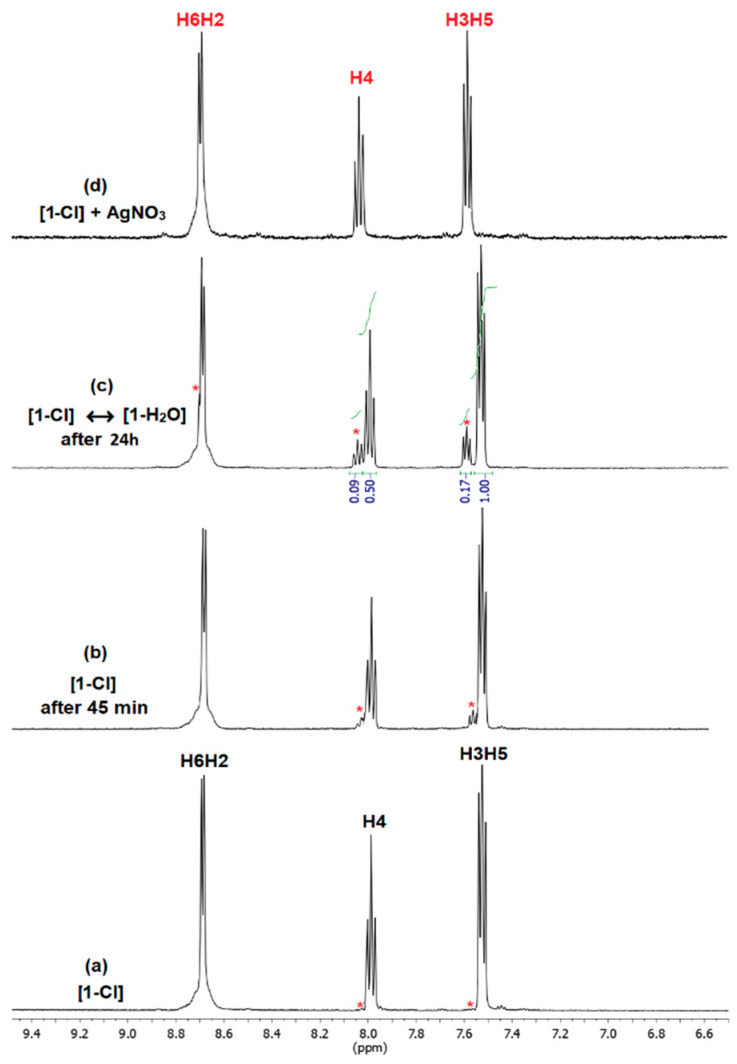
Aromatic region of the ^1^H NMR spectra of (**1**) in D_2_O at 298 K showing pyridine proton assignments. The red asterisks denote the aquated species. (**a**) The spectrum of (**1**) immediately upon dissolution in D_2_O. (**b**) The same sample of (**1**) after incubation at 298 K for 45 min. (**c**) The spectrum after 24 h at equilibrium. (**d**) The spectrum of (**1**) after the addition of one equivalent of AgNO_3_, precisely matching the signals of the aquated species.

To obtain the complete hydrolyzed spectrum of [Pt(en)(py)Cl]NO_3_ ([**1**-Cl]) as a reference, we performed its reaction with one equivalent of AgNO_3_. After removing the precipitated AgCl, the ^1^H NMR spectrum of the remaining solution was attributed to [Pt(en)(py)(OD_2_)]NO_3_ ([**1**-D_2_O]) (Figure 2d). Then, we monitored the hydrolysis of [**1**-Cl] in D_2_O at 298 K, recording ^1^H NMR spectra at proper time intervals. Upon dissolving [**1**-Cl] in D_2_O, the hydrolysis reaction started immediately, gradually altering the spectra towards the formation of the hydrolyzed product [**1**-D_2_O] (Figure 2). The kinetic of this reaction was slow in the NMR time scale and at 298 K, resulting in the appearance of new signals in the spectra. Quantification of the [**1**-D_2_O] fraction was achieved by integrating specific signals of the spectra. After 24 h, the hydrolysis reached equilibrium, yielding approximately 14.5% of the hydrolyzed product, while the spectrum remained unchanged even after several days. This percentage corresponds to an equilibrium constant, K_eq1_ = 0.079 mM at 298 K (Section 3.5, Equations (7) and (8)), which is similar to that observed for the hydrolysis of pyriplatin and phenanthriplatin (0.1 mM at 310 K) [12]. A slightly lower K_eq2_ value (K_eq_ = 0.071 mM) was calculated for (**2**), reflecting the difference between the pyridine and 2-methyl pyridine ligands.

**Figure 3:**
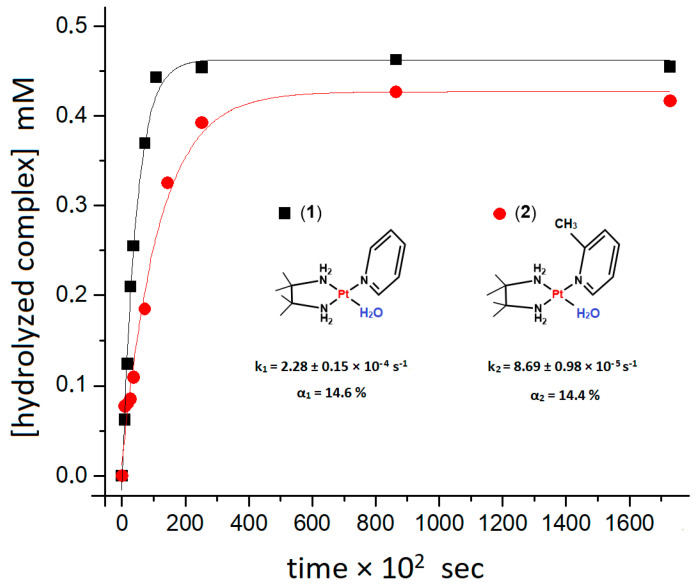
Plot showing the evolution concentrations of [**1**-D_2_O] and [**2**-D_2_O] over time during the hydrolysis of a solution of 3.2 mM of (**1**) (black squares) and 2.9 mM of (**2**) (red circles) in D_2_O, at 298 K, fitted with the pseudo-first-order kinetic Equation (9).

The aquation rates of the complexes (**1**) and (**2**) were investigated using ^1^H NMR spectroscopy, at 298 K in D_2_O in 3.2 mM and 2.9 mM solutions, respectively. Tables S1 and S2 illustrate the concentration of the [Pt(en)(py)(OD_2_)](NO_3_)_2_ ([**1**-D_2_O]) and Pt(en)(2-mepy)(OD_2_)](NO_3_)_2_ ([**2**-D_2_O]) as functions of time (t), while Figure 3 displays the corresponding plots. Since these reactions follow pseudo-first-order kinetics, we simulated them with the relevant Equation (9) (Section 3.6), yielding aquation rate constants for (**1**) k_(**1)**_ = 2.28 ± 0.15 × 10^−4^ s^−1^ and (**2**) k_(**2)**_ = 8.69 ± 0.98 × 10^−5^ s^−1^.

Complex (**1**) is hydrolyzed faster than (**2**), indicating an inhibition in the hydrolysis process due to the axially positioned methyl group of 2-mepy. In general, aquation rates are measured using different methods and under varying conditions of temperature and ionic strength; therefore, direct comparisons are challenging. However, an approximate comparison to similar complexes reveals that complex (**1**) is hydrolyzed faster than phenanthriplatin (0.62 ± 0.04 × 10^−4^ s^−1^ at 310 K), while (**2**) exhibits a similar hydrolysis rate. Regarding the complexes [Pt(dien)Cl]NO_3_ (6.50 ± 0.1 × 10^−5^ s^−1^ at 293 K) and [Pt(NH_3_)_3_Cl]NO_3_ (1.10 ± 0.03 × 10^−5^ s^−1^ at 293 K), which have a similar coordination sphere, complex (**1**) is hydrolyzed faster, while (**2**) is hydrolyzed similarly to [Pt(dien)Cl]NO_3_ [29,30].

**Figure 4:**
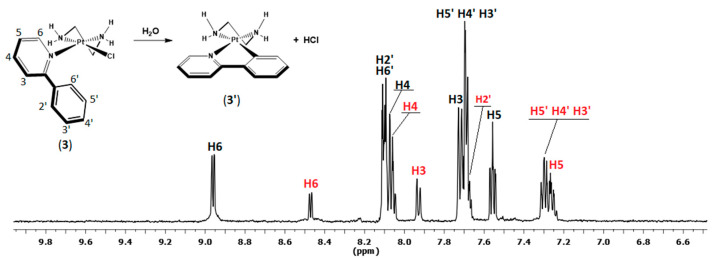
^1^H NMR spectrum of (**3**) (3 mM in D_2_O), showing the equilibrium mixture of [Pt(en)(phpy)Cl]^+^ and [Pt(en)(phpy-H^+^)]^+^ (**3′**), with proton assignments. The assignment in red was attributed to (**3′**).

The case of the nitrate salt complex (**3**), which possesses the phenyl ring substitution in the 2-position of the pyridine, was different to that of (**1**) and (**2**). Upon the dissolution of (**3**) in D_2_O, the ^1^H NMR spectra of (**3**) showed new signals assignable to the complex [Pt(en)(phpy-H^+^)]^+^, (**3′**). This suggests an alteration of the 2-phpy coordination, from monodentate to bidentate, accompanied by the deprotonation of C6′ and the subsequent formation of a Pt-C bond. The released [Cl]^−^ hindered the complete transformation of (**3**) to (**3′**), reaching equilibrium after 3 days in D_2_O at 298 K with Keq = 0.385 mM (Figure 4). The in situ characterization of (**3′**) was based on the COSY spectrum of the mixture, as well as in the HR-ESI-MS (Figure S3).

### 2.3. Interactions of (***1***) and (***2***) with Guanosine (Guo)

**Figure 5:**
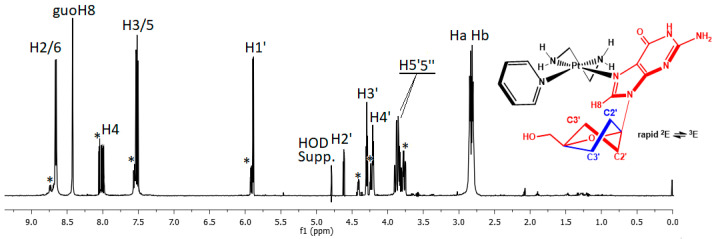
^1^H NMR spectrum of the 1:1 mixture of [**1**-D_2_O] and guanosine after 4 days incubation at 298 K. The reaction was completed by about 69%. Asterisks denote the signals of free guanosine and the complex [**1**-D_2_O]. Inset of the proposed structure of the complex [**1**-guo].

The interactions of complexes (**1**) and (**2**) with the nucleoside guanosine were studied using ^1^H NMR spectroscopy. Due to the complexity of the spectrum in the case of (**3**), we chose the simpler molecule of 9-methylguanine (9-MeG) as a DNA model. For complexes (**1**) and (**2**), one equivalent of AgNO_3_ was added to facilitate the formation of their aquated species, [**1**-D_2_O] and [**2**-D_2_O]. Subsequently, one equivalent of guanosine was added, and the spectra were recorded at appropriate time intervals for up to 4 days, keeping the sample at 298 K. During this time, the reaction of complex (**1**) was completed by about 69%, while at the case of (**2**) it reaches 95%, as determined using the ^1^H NMR spectra of the reaction mixtures. The results are shown in Figure 5 and Table 1. 

In the ^1^H NMR spectrum of the mixture, in addition to the signals of the remaining [**1**-D_2_O] and free guo, a new set of signals was observed, attributed to the complex [**1**-guo]. The signals of the pyridine ligand in this complex appeared slightly upfield (0.02–0.05 ppm), while the ethylenediamine Ha1a2 and Hb1b2 shifted downfield by 0.12 and 0.13 ppm, respectively, reflecting the change in the Pt(II) coordination sphere. Additionally, a singlet at 8.44 ppm, which was assigned to guoH8, shifted downfield by 0.41 ppm compared to free guanosine, indicating coordination through guoN7 [31].

The appearance of only one set of signals for [**1**-guo] in the ^1^H NMR spectrum of the reaction mixture could be attributed to three factors: (a) to the high symmetry of pyridine, (b) to the fast and unhindered rotation of the guanosine around the Pt-N7 bond at the NMR time scale at 298 K, or (c) to the formation of only one favored isomer, stabilized through the hydrogen bond between the guoO6 and one of the ethylenediamine amino groups [32]. Rapid rotation around the Pt-N7 bond of 9-RG (9-RG = 9-alkylguanine) prevents the discrimination of rotational isomers in the case of *cis*-[Pt(NH_3_)_2_(phenanthridine)(9-RG)]^2+^, even though a stable isomer through the interaction between the guoO6 and the *cis*-coordinated ammine has been suggested [32]. The guanosine sugar protons H1′, H4′, H5′, and H5′’ shifted slightly in the range of 0.02 to 0.03 ppm, while both the H2′ and H3′ shifted significantly upfield by 0.13 ppm, indicating an alteration in the relative populations of ^2^E (C_2′_*-endo*) and ^3^E (C_3′_*-endo*) conformers of the guo sugar (Figure 5). The ^13^C NMR spectrum of the mixture in D_2_O exhibited three sets of signals arising from (i) the free guo, (ii) the remaining [**1**-D_2_O], and (iii) the complex [**1**-guo]. Insignificant Δδ values were observed for the pyridine and ethylenediamine signals between the complexes [**1**-D_2_O] and [**1**-guo]. However, pronounced differences in ^13^C signals were observed between the coordinated guo and the free one. The most affected carbon was C8, neighboring the platinated N7, which shifted by Δδ = +1.3 ppm. In parallel, remarkable shifts were observed for the sugar carbons: C1′ (Δδ = +1.2 ppm), C2′ (Δδ = +0.3 ppm), and C3′ (Δδ = −0.7 ppm), indicating significant changes in sugar conformation, consistent with the observations in the ^1^H NMR spectrum (Table S3).

**Figure 6:**
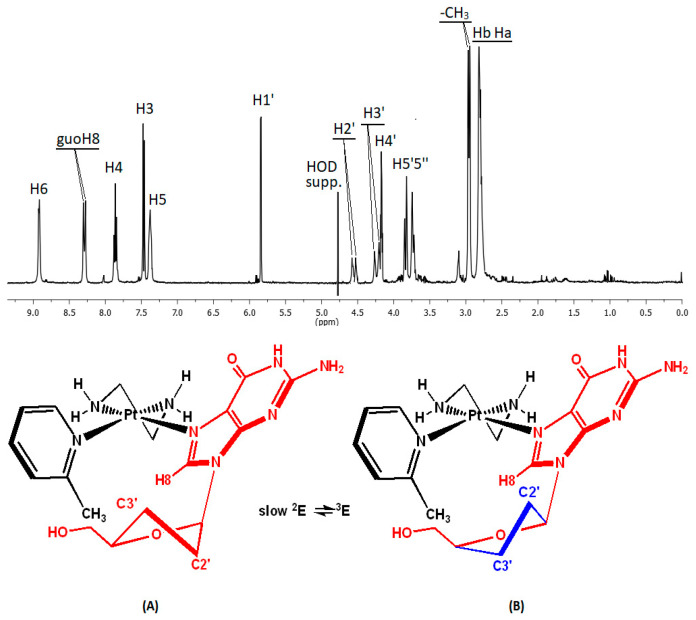
In the upper part, the ^1^H NMR spectrum of the 1:1 mixture of [**2**-D_2_O] and guanosine after 4 days incubation at 298 K, with the reaction completed by about 95%. At the bottom, the proposed isomers (**A**) and (**B**) of the complex [**2**-guo].

In the case of complex (**2**), insignificant shifts were observed for the 2-mepy protons, while the signals of H6 and H5 were notably broadened. In addition, the signal of pyridine -CH_3_ appeared as two distinct singlets, shifted upfield by 0.11 and 0.13 ppm (Figure 6). A double set of signals was also observed for most of the guanosine protons. GuoH8 appeared as two singlets at 8.31 and 8.29 ppm, and shifted less downfield compared to [**1**-guo], probably due to higher shielding from the 2-methylpyridine aromatic ring. Despite the insignificant difference between these signals (0.02 ppm), the downfield shift is large enough (Δδ = 0.26–0.28 ppm) to conclude coordination through N7. Similar results have been reported by Ma et al. in a study examining the binding of the complex [Pt(en)(PICAC)]NO_3_ (PICAC = 6-(methylpyridine-2-yl)acetate) with 5′-GMP, where the guoH8 exhibited two distinct, downfield-shifted signals, which, however, were very close (Δδ_H8_ = 0.02 ppm) [33]. This minimal difference observed in the NMR signals of H8 suggests that the formation of rotamers around the Pt-N bond is unlikely. Marzilli and co-workers have documented rotational isomers in the complex [Pt(L)(G)]^+^ {L = the tridentate ligand N-(6-methyl-2-picolyl)-N-(2-picolyl)amine and G = 9-ethylguanine, 3′-GMP, 5′-GMP, 5′-GTP}, with an unusually high abundance of the one rotamer [34]. However, the observed difference between the chemical shift of H8 in those rotamers was significant (Δδ_H8_ = 0.3–0.4 ppm), depending on the relative positioning of H8 toward the aromatic rings of the tridentate ligand.

Insignificant shifts and single signals were observed for the guanosine sugar protons, H1′, H4′, H5′, and H5′’. In contrast, H2′ and H3′ exhibited double signals and shifted significantly upfield (in the range of 0.13–0.21 ppm), indicating discernible sugar conformations. The pronounced shifts for the H2′ and H3′ suggest a slow equilibrium of the sugar ^2^E (C2′*-endo*) and ^3^E (C3′*-endo*) conformers, evident at 298 K in the NMR time scale. Considering the rapid equilibrium ^2^E ↔ ^3^E in the case of [**1**-guo], we can conclude that the presence of the -CH_3_ of 2-mepy impedes the swift transition between ^2^E and ^3^E of the guanosine sugar moiety, resulting in the observation of both conformers. This explanation also accounts for the broad signals of H5 and H6, as well as the double signals of the methyl group of 2-mepy. In addition, the slight difference observed in the signals of H8 could also be attributed to the presence of two guo-sugar conformers, rather than the orientation of guoH8 toward the methyl group of 2-mepy. In the ^13^C NMR spectrum of the reaction mixture in D_2_O, we observed one set of ^13^C signals corresponding to 2-mepy and ethylenediamine. The signals of the guanosine C8 and the sugar carbons C2′ and C3′ appeared to be double, with shifts observed for C8 (Δδ = +1.3/+1.1 ppm), C2′ (Δδ = −0.3/−0.4 ppm), and C3′ (Δδ = −0.8/−0.7 ppm).

**Figure 7:**
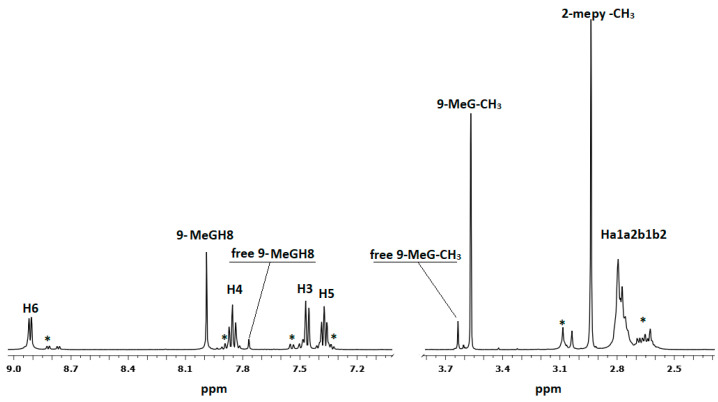
^1^H NMR spectrum (D_2_O and 298 K) of [**2**-(9-MeG)], showing the single signals of the N7-coordinated 9-MeGH8, 9-MeG-CH_3_, and the 2-mepy-CH_3_. Asterisks denote the signals of the complex [**2**-D_2_O].

To further support the claim that the presence of two isomers in [**2**-guo] is attributed to the sugar unit of guanosine, we conducted the same reaction under identical conditions using 9-MeG. Unlike guanosine, 9-MeG possesses a methyl group at the nucleobase N9 position, minimizing steric hindrance with 2-mepy. In contrast to our observation in [**2**-guo], the resulting spectrum for [Pt(en)(2-mepy)(9-MeG)](NO_3_)_2_ {[**2**-(9-MeG)]} displayed only one single resonance for 9-MeGH8 (Δδ = +0.23 ppm) and 9-MeGCH_3_ (Δδ = −0.07 ppm), with the other proton signals of the complex remaining almost unaffected (Figure 7). This observation indicates that the presence of two isomers in [**2**-guo] arises from the slow transition C2′-*end*o ↔ C3′-*endo* of the guanosine sugar moiety. Thus, the hypothesis that the methyl group of 2-mepy impedes the swift transition C2′-*end*o ↔ C3′-*endo* in (**2**) is confirmed.

#### Sugar Conformation Analysis

Aiming to better understand the effects of the binding of (**1**) and (**2**) to the DNA structural features, we utilized NMR spectroscopy to analyze the conformation of the guanosine sugar moiety, based on ^1^H-^1^H spin–spin coupling constants (^3^*J*) [35]. These local perturbations may impede DNA transcription and repair mechanisms. Previous studies suggested that the complex [Pt(NH_3_)_2_(py)Cl]^+^ induces less significant distortions to the DNA helix when binding to a guanine N7, in comparison to cisplatin [10]. Also, the X-ray crystal structure of [Pt(NH_3_)_2_(py)Cl], bound to a modified oligonucleotide, causes a duplex unwinding of 8°, whereas cisplatin causes a greater unwinding of the duplex by 13°.

Using established NMR methods [36,37,38,39,40,41], we conducted conformational analyses of the guanosine sugar moiety in cases (**1**) and (**2**). Through examination of the H2′ chemical shift, we determined the relative orientation of the sugar moiety in relation to the purine ring system (*syn ↔ anti*), a key factor influencing nucleic base spacing in DNA. It has been reported that nucleosides featuring bulky substituents at the C8 position of the purine ring hinder the *anti*-conformation by the sugar moiety, favoring the *syn*-. Such modified nucleosides, as 8-bromoguanosine (8-Brguo) [42], 8-N-acetyl-2-aminofluoreneguanosine (8-AAFguo) [43], and 8-tert butyl guanosine monophosphate (8-*^t^*BuGp) [44], induce downfield shifts in the H2′ signal of the sugar moiety. On the contrary, highly *anti-*conformations were observed in cases involving MgN7Gp or PtN7Gp coordination [45], leading to an upfield shift of H2′. Considering that the sugar is exclusively oriented in the *syn-*conformation in 8-*^t^*BuG (δ_H2_′ = 5.07 ppm) [44], while it is exclusively in the *anti-*conformation in the compound of N7 platinated guanosine *trans-*[Pt(guo)_2_(alaH)_2_]Cl_2_ (δ_H2_′ = 4.49 ppm) [41], an empirical equation can be proposed to determine the percentages of *syn-*and *anti-*conformers. This equation postulates that the degree of the change in the chemical shift of H2′ linearly corresponds to the changes in populations of the *syn-* and *anti-* conformers.

% anti = {(δ_H2′ 8-*t*BuGp_ − δ_H2′X_)/(δ_H2′ 8-*t*BuGp_ − δ_H2′trans−[Pt(guo)2(alaH)2]Cl2_)} × 100 (1)

% syn = 100 − % anti(2)

where δ_H2′X_ is the H2′ chemical shift of the studied compound.

The data presented in Table 2 reveals that the guanosine sugar moiety in both (**1**) and (**2**) exhibited a preference for the *anti-*conformation compared to free guanosine [46]. This aligns with the well-known effect of cisplatin binding to guoN7, i.e., the increase in the amount of *anti-*conformation [47]. Interestingly, [**2**-guo]A exhibits similarities to [**1**-guo], practically favoring the *anti-*conformation. On the other hand, the [**2**-guo]B adopts a more pronounced tendency for *anti-*conformation (93%), which may induce greater distortion in the DNA helix. 

**Figure 8:**
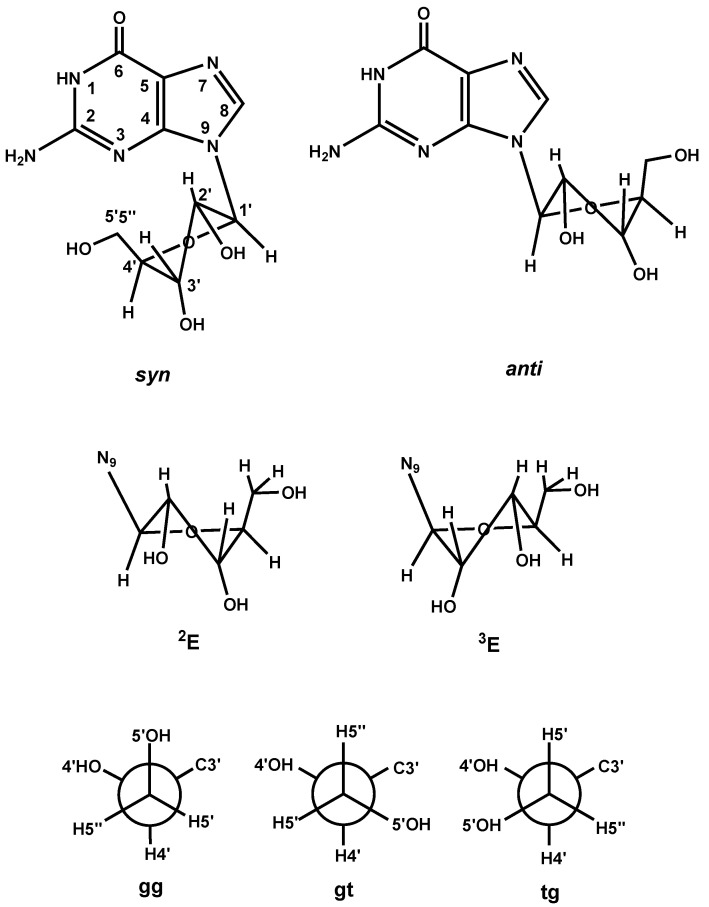
Sugar conformational isomers of guanosine, with structure numbering.

By analyzing the ^3^*J* coupling constant of the guanosine sugar protons and employing Equations (3) and (4), we determined the percentage of the ^3^E (C3′-*endo*) and ^2^E (C2′-*endo*) conformers (rapid equilibrium) of the five-membered sugar ring. Also, by utilizing Equations (5) and (6), we gained insight into the C4′–C5′ bond torsion angles, expressed as percentages of the population gg and (gt + tg) (Figure 8) [36,37,38,39,47,49].

% ^2^E = [*J*_1′2′_/(*J*_1′2′_ + *J*_3′4′_)] × 100 (3)

and

% ^3^Ε = 100 − %^2^Ε (4)

% gg = [(13 − Σ)/10] × 100, Σ = (*J*_4′5′_ + *J*_4′5″_) (5)

and

% (gt + tg) = 100 − %gg(6)

These structural features define the backbone conformation of DNA, suggesting alterations in the geometry of the helix [50]. An increase in the ^3^*E* guanosine sugar conformation is characteristic of N7-platination [48], N7-methylation [36], and N7-protonation [48]. 

In the case of [**1**-guo], the rapid equilibrium between the conformations ^3^E and ^2^E facilitated the determination of their relative populations in the solution, as illustrated in Table 2. This rapid equilibrium suggests a fast transition between the conformers at 298 K and in the NMR time scale. In contrast, for [**2**-guo], it is assumed that a slow equilibrium exists between ^3^E and ^2^E, allowing for the distinction of the two conformers. This slow equilibrium is characterized by distinct signals for the involved sugar protons, and their relative populations may be calculated only from their signal integrals, resulting in nearly equal percentages. Regarding the gg conformation about the bond C4′–C5′, although a decrease in the gg percentage was expected [48], it appears to be practically stable for [**1**-guo] and [**2**-guo]A, while it is slightly lower for [**2**-guo]B. In other words, the platination of guo by (**1**) and (**2**) does not practically affect the conformation around the C4′–C5′ bond compared to free guanosine. It is notable that a dramatic increase in the gg conformer has been reported in cases of N7 methylation or protonation of Gp5′ (Table 2).

### 2.4. Interactions of (***3***) with 9-Methyl Guanine (9-MeG)

**Figure 9:**
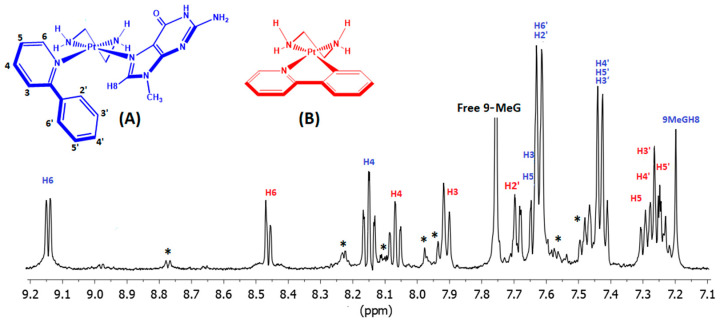
^1^H NMR of the reaction mixture of (**3**) with 9MeG in D_2_O (5 mM NaCl) at 310 K. In red color, the assignments of (**3′**), structure **B**, and in blue color, the assignments of the complex [Pt(en)(2-phpy)(9-MeG)]^2+^, structure **A**. Inset of the corresponding proposed structures. Minor products (<10%) indicated with asterisks (*).

To inhibit the fast hydrolysis of (**3**), its interaction with 9-MeG was studied in 5 mM NaCl. Under these conditions, a solution containing 5 mM of (**3**) was treated with a four-fold excess of 9-MeG. The ^1^H NMR spectrum of the mixture was recorded at appropriate time intervals up to 4 days at 310 K. After 4 days, the spectrum remained practically unchanged, indicating that the system has reached equilibrium (Figure 9).

In the equilibrium state, mainly (~90%) two distinct species were detected, which were identified with the assistance of 2D COSY, NOESY, and HSQC experiments. Despite the presence of 5 mM NaCl, (**3**) is partially transformed to (**3′**), and this species is depicted in red color in Figure 9. Its ^1^H NMR signals perfectly matched those of (**3′**), observed during the hydrolysis of (**3**) (Figure 4).

Furthermore, another major species was observed involving the coordination of 9-MeG, indicated by the blue color in Figure 9, at a 1:1 molar ratio, as calculated from selected proton signal integrals. The [Pt(en)(2-phpy)(9-MeG)]Cl_2_ {[**3**-(9-MeG)]} species can be formulated as [Pt(en)(2-phpy)(9-MeG)]^2+^. Considering the acidic environment of the reaction mixture (pD ≈ 5) resulting from the release of HCl during the ring closure of 2-phpy, 9-MeG is expected to coordinate through N7. However, the signal at 7.21 ppm, assigned to 9-MeGH8 based on HSQC cross-peak with 9-MeGC8 (141.2 ppm), exhibited a significant upfield shift (Δδ = −0.55 ppm), indicative of a rich electron density environment around H8. In this case, a proposed structural arrangement positions the 9-MeGH8 above the phenyl ring of 2-phpy, resulting in significant shielding of the 9-MeGH8 (Figure 9 and Figure S10, and Table 3).

**Figure 10:**
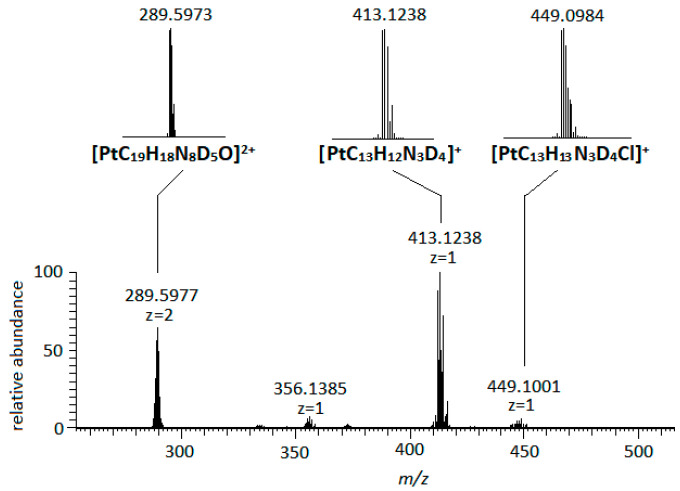
The positive mode of the HR-ESI mass spectrum of the reaction mixture containing (**3**) and 9-MeG. Inset of the calculated cluster-peaks of the cations at *m/z* = 289.5977, 413.1238, and 449.1001.

The presence of the aforementioned species was further confirmed through mass spectrometry. In the HR-ESI-MS of the reaction mixture in D_2_O, a cluster-peak at *m/z* = 413.1238 was attributed to the single-charged cation [PtC_13_H_12_N_3_D_4_]^+^, corresponding to (**3′_D4_**). This assignment assumes that the labile protons of the ethylenediamine amino groups were exchanged with deuterium from D_2_O. Similarly, a weak intensity cluster-peak at *m/z* = 449.1001 was assigned to the cation [PtC_13_H_13_N_3_D_4_Cl]^+^, (**3_D4_**), while the cluster peak at *m/z* = 289.5977 was assigned to the double-charged cation [PtC_19_H_18_N_8_D_5_O]^2+^. The latter was attributed to the 1:1 complex of (**3**) with 9-MeG formulated as [Pt(en)(2-phpy)(9-MeG)]^2+^. In this cation, a labile proton of the 9-MeG was exchanged with a deuterium atom from the solvent. The calculated spectra for these cluster-peaks exhibited excellent agreement with the experimental *m/z* values and the isotopic distributions (Figure 10).

### 2.5. Cytotoxic Activity

**Figure 11:**
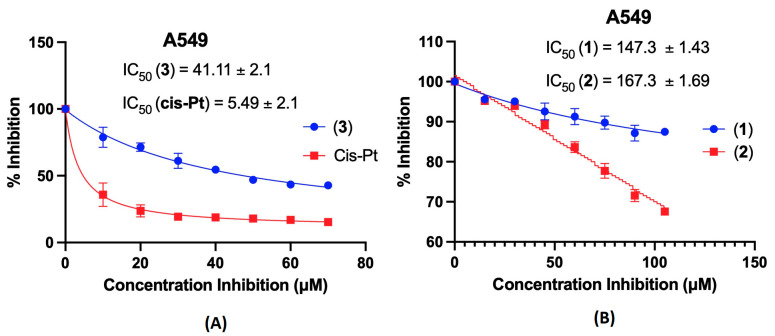
(**A**) Plot of the % cell viability vs. the concentrations of (**3**) and cisplatin against the human lung cancer cell line A549. (**B**) Plot of the % cell viability vs. the concentrations of (**1**) and (**2**). Results represent the average of three independent experiments.

The in vitro cytotoxicity of the complexes (**1**)–(**3**) was evaluated against the human A549 lung cancer cell line over a 48-h incubation period. Complex (**3**) demonstrated significant efficacy, displaying an IC_50_ value of 41.11 ± 2.1 μM (Figure 11). In contrast, complexes (**1**) and (**2**) were almost inactive, exhibiting IC_50_ values > 100 μM. It is worth mentioning that both complexes (**1**) and (**2**) exhibited higher IC_50_ values compared to analogous complexes *cis*-[Pt(NH_3_)_2_(py)Cl]NO_3_ (IC_50_ = 52.1 ± 2.3 μM) and *cis*-[Pt(NH_3_)_2_(2-mepy)Cl]NO_3_ (IC_50_ = 50.6 ± 1.7 μM), indicating that replacing the two *cis*-amino groups with ethylenediamine has a negative effect on the cytotoxicity of such complexes [12]. Also, complex (**1**) was reported to be almost inactive (IC_50_ > 300 µM) against the human DLD-1 colorectal adenocarcinoma tumor cell line [51]. Furthermore, the efficacy of complex (**3**) with IC_50_ = 41.11 ± 2.1 μM against the A549 cancer line highlights the significance of a bulky shaped auxiliary ligand in the antitumor potency of monofunctional platinum complexes. More specifically, the 2-substitution of pyridine with phenyl enhances cytotoxicity in (**3**) compared to both its corresponding methyl substitution in (**2**) and unsubstituted pyridine in (**1**). Other monofunctional analogues of pyriplatin complexes have shown similar results. In particular, a series of platinum complexes with BODIPY (boron-dipyrromethene)-conjugated pyridine showed up to 1200 times greater photocytotoxicity against the A549 cancer cell line compared to the analogous complex with 4-methylpyridine [52]. Also, the findings by Zhu et al. confirm that an increase in pyridine bulkiness enhances cytotoxicity, as they reported that complexes of pyriplatin analogs with methylenetriphenylphosphorane-substituted pyridine inhibited A549 cells more effectively compared to pyriplatin [24]. In addition to the primary requirement for a bulky ligand, numerous other factors contribute to the antitumor activity of monofunctional platinum complexes. Although the complexes (**1**)–(**3**) share common properties with phenanthriplatin, such as DNA-binding characteristics, hydrolysis rates, and equilibrium constants, phenanthriplatin is significantly more cytotoxic with a IC_50_ value of 0.22 ± 0.01 µM against A549 cells [5,12].

## 3. Materials and Methods

### 3.1. Chemicals

All solvents were of analytical grade, and were used without further purification. Potassium tetrachloroplatinate(II) K_2_[PtCl_4_] (99.9%), ethylenediamine, pyridine, 2-methylpyridine, 2-phenylpyridine guanosine, and 9-methylguanine were purchased from Alfa Aesar (Haverhill, MA, USA). The complex [Pt(en)Cl_2_] was synthesized according to the literature methods [53,54].

### 3.2. Methods

^1^H NMR spectra were recorded onBruker NEO spectrometer (Bruker, Billerica, MA, USA) operating for ^1^H frequencies of 400.13 and 500.13 MHz, and were processed using Topspin 4.2 (Bruker Analytik GmbH, Bruker, Billerica, MA, USA). COSY experiments were used to assist the assignments of ^1^H signals. High resolution electrospray ionization mass spectra (HR-ESI-MS) were obtained on an Agilent Technology LC/MSD trap SL instrument and Thermo Scientific, LTQ Orbitrap XL™ system (Waltham, MA, USA). C, H, and N determinations were performed on a Perkin-Elmer 2400 Series II analyzer (Waltham, MA, USA). The IR spectra of the complexes were recorded on an Agilent Cary 630 FTIR spectrometer (Santa Clara, CA, USA) (Figures S7–S9).

### 3.3. Cell Culture

The human lung adenocarcinoma alveolar basal epithelial cells A549 were cultured in Dulbecco’s modified Eagle’s medium (DMEM, high glucose) supplemented with 1% glutamine and 1% penicillin/streptomycin. Cells were routinely passaged every 2 or 3 days, and were incubated in a humidified atmosphere of 5% CO_2_ at 37 °C. The medium was changed every other day. When the cells reached confluence, they were detached using 0.2% (*w*/*v*) trypsin and were transferred to new culture flasks. Viability was routinely maintained at >95%, as assayed using the Crystal Violet exclusion method.

### 3.4. Cell Growth Assay

The A549 tumor cells were treated with increasing concentrations of complexes (**1**), (**2**), (**3**), and cisplatin for 48 h, and were subsequently stained with crystal violet dye. The Tecan Plate Reader Infinite 200 M Pro (Männedorf, Switzerland)and its software were used to monitor the cell growth and verify the cytotoxicity effects of the complexes. The IC_50_ values of the complexes and of cisplatin were determined by fitting the inhibitor concentration versus the response curve using Graphpad Prism version 9.5.1.

### 3.5. Determination of Equilibrium Constants

The equilibrium constants (K_eq_) for the hydrolysis reactions of the complexes (**1**) and (**2**) were determined by monitoring their ^1^H NMR spectra at 298 K of a freshly prepared solutions with initial concentrations (C_i_) of 3.2 mM and 2.9 mM, respectively. The equilibrium fraction (F_eq_) of [**1** or **2**-H_2_O] was estimated after the hydrolysis reaction reaches the equilibrium measuring the integral ratio (r_eq_) of selected signals of [**1** or **2**-Cl] and [**1** or **2**-H_2_O], according to Equation (7) [55], as follows:

F_eq_ = r_eq_/(1 + r_eq_) (7)

The equilibrium constants at 298 K for (**1**) and (**2**) were calculated using the Equation (8), as follows:

K_eq_ = [F_eq_C_i_]^2^/[(1 − F_eq_)C_i_] (8)

### 3.6. Determination of Hydrolysis Constants

The hydrolysis reaction of (**1**) and (**2**) was monitored using ^1^H NMR spectroscopy in D_2_O at 298 K, with concentrations of 3.2 mM and 2.9 mM, respectively. The quantification of the [**1** or **2**-D_2_O] was obtained using Equation (7) for fractions (F_t_) at a specific time t (s). Plots of the aquated fractions (F_t_) versus time were fitted using a pseudo-first order kinetic, Equation (9), to give the rate constants k_(**1**)_ and k_(**2**)_ [55], as follows:

A = A_1_ − A_2_(e^−kt^) (9)

A_1_ and A_2_ are constants related to the initial and equilibrium concentration of the complex.

### 3.7. Synthesis of the Complexes (***1***)–(***3***)

The complexes (**1**) and (**2**) were synthesized in a similar way.

In a typical experiment, 0.1 mmol of [Pt(en)Cl_2_] was dissolved in 3 mL of DMF in a 10 mL a vial, followed by the addition of 0.095 mmol of AgNO_3_. The mixture was left at room temperature overnight in the dark, and the precipitated AgCl was removed using centrifugation. To the supernatant solution, 0.12 mmol of L (pyridine or 2-methyl pyridine) was added, and the mixture was then heated at 55 °C overnight. The resultant solution was transferred into a round-bottomed flask and evaporated to dryness under reduced pressure. Subsequently, 2 mL of MeOH was added, and any insoluble unreacted materials were removed using centrifugation. To the remaining solution, a 25 mL of a mixture (4:1, *v*/*v*) diethyl ether: acetone was added, affording a white solid which was collected using filtration, washed with diethyl ether, and dried in a vacuum over P_2_O_5_. 

**[Pt(en)(py)Cl]NO_3_, (1)**: Yield 55%. Anal. for C_7_H_13_ClN_4_O_3_Pt, cal., C: 19.47%; H: 3.04%, N: 12.98%, found, C: 19.30%, H: 3.12%, N: 12.91%. HR-ESI-MS, positive (*m/z*), found, 369.0430, calc. 369.0440 for [C_7_H_13_ClN_3_^195^Pt]^+^. ^1^H NMR (500 MHz, 298K, D_2_O, δ in ppm): H2/6: 8.70 (d, 2H, *^3^J_H-H_* = 6.2 Hz), H4: 8.00 (t, 1H, *^3^J_H-H_* = 7.8 Hz), H3/5: 7.54 (t, 2H, *^3^J_H-H_* = 7.2 Hz), Ha1a2 and Hb1b2: 2.72 (m, 2H) and 2.67 (m, 2H). 

**[Pt(en)(2-mepy)Cl]NO_3_, (2)**: Yield 80%. Anal. for C_8_H_15_ClN_4_O_3_Pt, cal., C: 21.56%; H: 3.39%, N: 12.57%, found, C: 21.45%; H: 3.39%, N: 12.60%. HR-ESI-MS, positive (*m/z*), found, 383.0604, calc. 383.0597 for [C_8_H_15_ClN_3_^195^Pt]^+^. ^1^H NMR (400 MHz, 298 K, D_2_O, δ in ppm): H6: 8.80 (d, 1H, *^3^J_H-H_* = 5.95 Hz), H5: 7.37 (t, 1H, *^3^J_H-H_* = 7.1 Hz), H4: 7.86 (t, 1H, *^3^J_H-H_* = 7.8 Hz), H3: 7.52 (d, 1H, *^3^J_H-H_* = 7.8 Hz), HCH_3_: 3.09 (s, 3H), Ha1a2 and Hb1b2: 2.71 (m, 2H) and 2.66 (m, 2H).

**[Pt(en)(2-phpy)Cl]Cl, (3)**: The complex (**3**) was synthesized similarly to (**1**) and (**2**), but the compound required further purification. Thus, the crude product was purified with column chromatography using a 1:1 mixture of MeOH:MeCN saturated with NH_4_PF_6_ as the eluent. The resulting fraction was evaporated to 1 mL under reduced pressure, and 25 mL of a mixture (4:1, *v*/*v*) diethyl ether: Acetone was added to form a pale yellow solid. After cooling the mixture overnight at 4 °C, the solid was collected using filtration, washed with diethyl ether, and dried in a vacuum over P_2_O_5_. The complex was isolated as [PF_6_]^−^ salt which was transformed to its corresponding water-soluble [Cl]^−^ salt, as described earlier [56]. Yield 26%. Anal. for C_13_H_17_Cl_2_N_3_Pt, cal., C: 32.44%; H: 3.56%, N: 8.73%, found, C: 32.47%; H: 3.57%, N: 8.69%. HR-ESI-MS, positive (*m/z*), found, 445.0753, calc. 445.0753 for [C_13_H_17_ClN_3_^195^Pt]^+^. ^1^H NMR (500 MHz, 298 K, D_2_O, δ in ppm): H6: 8.96 (d, 1H, *^3^J_H-H_* = 5.8 Hz), H2′H6′: 8.10 (d, 2H, *^3^J_H-H_* = 7.4 Hz), H4: 8.07 (t, 1H, *^3^J_H-H_* = 7.9 Hz), H3: 7.72 (t, 1H, *^3^J_H-H_* = 7.7 Hz), H3′H4′H5′: 7.68 (m, 3H), H5: 7.55 (t, 1H, *^3^J_H-H_* = 6.8 Hz), Ha1a2 and Hb1b2: 2.46 and 2.41 (m, 4H). 

To study the hydrolysis of (**3**), 1 equivalent of AgNO_3_ was added to an aqueous solution of (**3**), and the resulting AgCl was removed using filtration. The in situ formation of the nitrate salt of (**3**) was confirmed using mass spectrometry (*m/z* = 445.0740). The ^1^H NMR spectrum of [Pt(en)(2-phpy)Cl]NO_3_ slightly differs from the original spectrum of (**3**).

## 4. Conclusions

Classical Pt(II) drugs, such as cisplatin, carboplatin, and oxaliplatin, which are used in cancer chemotherapy, face limitations due to cellular drug resistance mechanisms. These drugs form 1,2-GG cross-links with DNA, triggering repair mechanisms like NER (nucleotide excision repair) [57]. To avoid this, minimal structural changes are needed to induce cell apoptosis without activating NER. Resistance mechanisms involving glutathione also pose a challenge, as they remove the drug from the cell by forming stable Pt-S bonds. Monofunctional Pt complexes, such as phenanthriplatin [5,12], are proposed to address these issues by causing fewer DNA structural changes and slowing Pt-S bond formation. 

Studying a series of complexes, (**1**)–(**3**), with the general formula [Pt(en)(L)Cl]^+^ (L is 2-substituted pyridine with substituents, H, Me, Ph), we observed that the hydrolysis rate of [Cl]^−^ decreases with the bulkiness of the substitution for (**1**) and (**2**). The rate values followed the trend that the bulkier the substitution, the slower the hydrolysis (k_(**1**)_ = 2.28 ± 0.15 × 10^−4^ s^−1^ and k_(**2**)_ = 8.69 ± 0.98 × 10^−5^ s^−1^ at 298 K). While these values align with the value of the phenanthriplatin (0.62 ± 0.04 10^−4^ s^−1^ at 310 K), (**1**) and (**2**) do not exhibit significant cytotoxicity (IC_50_ > 100 μΜ). Interestingly, the similar complexes *cis-*[Pt(NH_3_)_2_(py)Cl]NO_3_ and *cis-*[Pt(NH_3_)_2_(2-mepy)Cl]NO_3_ display higher cytotoxicity against the A549 cancer cell line, highlighting the importance of having two *cis-*ammino groups in the platinum coordination sphere, instead of ethylenediamine. Complex (**3**) behaves differently than (**1**) and (**2**). Upon hydrolysis, an equilibrium (K_eq_ = 0.385 mM) between the complexes [Pt(en)(2-phpy)Cl]^+^ and [Pt(en)(2-phpy-H^+^)]^+^ was observed, while there is no evidence (NMR or HR-ESI-MS) for the presence of the aquated complex [Pt(en)(2-phpy)(H_2_O)]^2+^. However, (**3**) exhibits good cytotoxicity (IC_50_ = 41.11 ± 2.1 μΜ), likely due to the interaction of the species [Pt(en)(2-phpy)Cl]^+^ with DNA.

Regarding the DNA binding of (**1**) and (**2**) to the DNA-model guanosine, we confirmed their binding through guoN7, which increased the population of the ^3^E sugar conformation. However, we observed that the rapid transition ^2^E ↔ ^3^E, in the case of [**1**-guo], is slower in the case of [**2**-guo], resulting in distinct signals for the two conformations in the ^1^H NMR spectra. Τhis phenomenon arises from a steric hindrance between the methyl group of pyridine and the sugar moiety of guanosine. However, this hindrance is absent in [**2**-(9-MeG)], which lacks the bulky sugar unit. 

In the case of (**3**), where the bulkiness of the substitution on the pyridine is further increased by a phenyl group, we observed a notable proximity of 9-MeGH8 to the phenyl ring of 2-phpy. Considering that only (**3**) exhibits good cytotoxicity against the A549 cancer cell line, we may conclude that ligands, L, with an extended aromatic system and proper orientation in complexes of the type *cis*-[Pt(en)(L)Cl]NO_3_ may enhance the cytotoxic activity of the complex.

## Figures and Tables

**Table 1:** ^1^H NMR chemical shifts (ppm) of the complexes [**1**-D_2_O], [**2**-D_2_O] and their adducts with guanosine and 9-MeG in D_2_O and at 298 K. In parentheses, the ^3^*J* coupling constants of the guanosine sugar protons (Hz).

Compound	Pyridine/2-Methylpyridine						en	Guanosine/9-Methylguanine							
	H2	H3	H4	H5	H6	CH_3_	Ha_1_a_2_/b_1_b_2_	H8	CH_3_	H1′ (*J*_1′2′_)	H2′ (*J*_2′3′_)	H3′ (*J*_3′4′_)	H4′	H5′ (*J*_4′5′_)	H5″ (*J*_4′5′’_)
guo								8.03		5.91 (5.95)	4.74 (5.40)	4.41 (3.80)	4.18	3.89 (3.05)	3.74 (4.05)
[**1**-D_2_O]	8.71	7.56	8.03	7.56	8.71	-	2.72–2.67								
[**1**-guo]	8.66	7.52	8.00	8.66	7.52	-	2.84–2.80	8.44		5.89 (4.85)	4.61 (4.95)	4.29 (5.60)	4.21	3.86 (2.75)	3.77 (4.15)
[**2**-D_2_O]	-	7.54	7.83	7.34	8.83	3.09	2.71–2.66								
[**2**-guo]A	-	7.48	7.87	7.39	8.93b	2.96	2.82–2.80	8.31		5.84 (4.70)	4.58 (4.30)	4.28 (5.60)	4.18	3.85 (2.75)	3.74 (4.95)
[**2**-guo]B	-	7.48	7.87	7.39b	8.93b	2.98	2.82–2.80	8.29		5.84 (4.70)	4.53 (4.60)	4.22 (4.90)	4.18	3.85 (2.75)	3.74 (4.05)
9-MeG								7.76	3.65						
[**2**-(9-MeG)]		7.46	7.86	7.37	8.91	2.96	2.81 2.79	7.99	3.58						

b = broad.

**Table 2:** Percentages (%) for guanosine sugar conformation in the complexes [**1**-guo], [**2**-guo]A, and [**2**-guo]B, as calculated from the Equations (1)–(6).

Compound	anti (%)	syn (%)	^3^E (%)	^2^E (%)	gg (%)	(gt + tg) (%)
guo	57	43	39	61	59	41
[**1**-guo]	79	21	54	46	61	39
[**2**-guo]A	85	15	-	-	62	38
[**2**-guo]B	93	7	-	-	53	47
Pt-N7Gp5′ ^a^			51	49	36	64
Me-N7Gp5′ ^b^			54	46	97	3
H^+^-N7Gp5′ ^a^			57	43	87	13
Gp5′ ^a^			36	64	65	35

^a,b^ Taken from reference [48] and [36], respectively.

**Table 3:** ^1^H NMR chemical shifts of the species observed in the reaction mixture of a solution containing (**3**) (5 mM in 5 mM NaCl) with 9-MeG in D_2_O at 310 K.

Compound	2-phpy									9-MeG		en
	H3	H4	H5	H6	H2′	H3′	H4′	H5′	H6′	H8	-CH_3_	Ha_1_a_2_/b_1_b_2_
9-MeG in D_2_O										7.76	3.58	
(**3**)	7.72	8.07	7.55	8.96	8.10	7.68	7.68	7.68	8.10	-	-	2.41–2.46
(**3**′)	7.92	8.07	7.25	8.46	7.69	7.32	7.32	7.31	-	-	-	2.58–2.56
[**3**-(9-MeG)]	8.23	8.15	7.65	9.12	7.64	7.44	7.44	7.44	7.64	7.21	3.62	2.60–2.55

## Data Availability

Data are contained within the article and Supplementary Materials.

## Supplementary Materials

The following supporting information can be downloaded at: https://www.mdpi.com/article/10.3390/ijms25126526/s1.
