# Supplementary material for: The Influence of the Auxiliary Ligand in Monofunctional Pt(II) Anticancer Complexes on the DNA Backbone
Source: Int J Mol Sci. 2024 Jun 13;25(12):6526. doi: 10.3390/ijms25126526 (PMC11203703; doi:10.3390/ijms25126526)
Supplement: Supplementary file 1 [file ijms-25-06526-s001.zip › ijms-3032918-supplementary.pdf]

Supplementary material

The influence of the auxiliary ligand in monofunctional Pt(II) anticancer complexes on the DNA backbone.

Evanthia-Vasiliki Tagari[1], Evangelia Sifnaiou[1], Theodoros Tsolis[1] and Achilleas Garoufis[1,2,*]

[1] Laboratory of Inorganic Chemistry, Department of Chemistry, University of Ioannina, 45110 Ioannina, Greece; ch05512@uoi.gr; e.sifnaiou@uoi.gr; t.tsolis@uoi.gr
[2] University Research Centre of Ioannina (URCI), Institute of Materials Science and Computing, Ioannina, Greece
* Correspondence: agaroufi@uoi.gr

Table of contents

Figure S1: HR-ESI-MS spectrum of the complex [Pt(en)(py)Cl]NO_3 (**1**) in H_2O at 298K.
Figure S2: HR-ESI-MS spectrum of the complex [Pt(en)(2-mepy)Cl]NO_3 (**2**) in H_2O at 298K.
Figure S3: HR-ESI-MS spectrum of the complex [Pt(en)(2-phpy)Cl]Cl (**3**) and [Pt(en)(2-phpy)]Cl (**3′**) in H_2O at 298K.
Figure S4: ^{1}H NMR of the complex [Pt(en)(py)Cl]NO_3 (**1**) inD_2O at 298K.
Figure S5: ^{1}H NMR of the complex [Pt(en)(2-mepy)Cl]NO_3 (**2**) in D_2O at 298K
Figure S6: ^{1}H NMR of the complex [Pt(en)(2-phpy)Cl]Cl (**3**) in D_2O at 298K.
Figure S7: IR spectrum of [Pt(en)(py)Cl]NO_3 (**1**) at 298 K.
Figure S8: IR spectrum of [Pt(en)(2-mepy)Cl]NO_3 (**2**) at 298K.
Figure S9: IR spectrum of [Pt(en)(2-phpy)Cl]Cl (**3**) at 298K.
Table S1: Concentrations of the hydrolyzed complex (**1**), [**1**-H_2O], as functions of time (t).
Table S2: Concentrations of the hydrolyzed complex (**2**), [**2**-H_2O], as functions of time (t).
Table S3: ^{13}C NMR data for selected species.
Figure S10: HSQC of the mixture of complex (**3**) and 9-methylguanine with NaCl 5 mM at 310K.

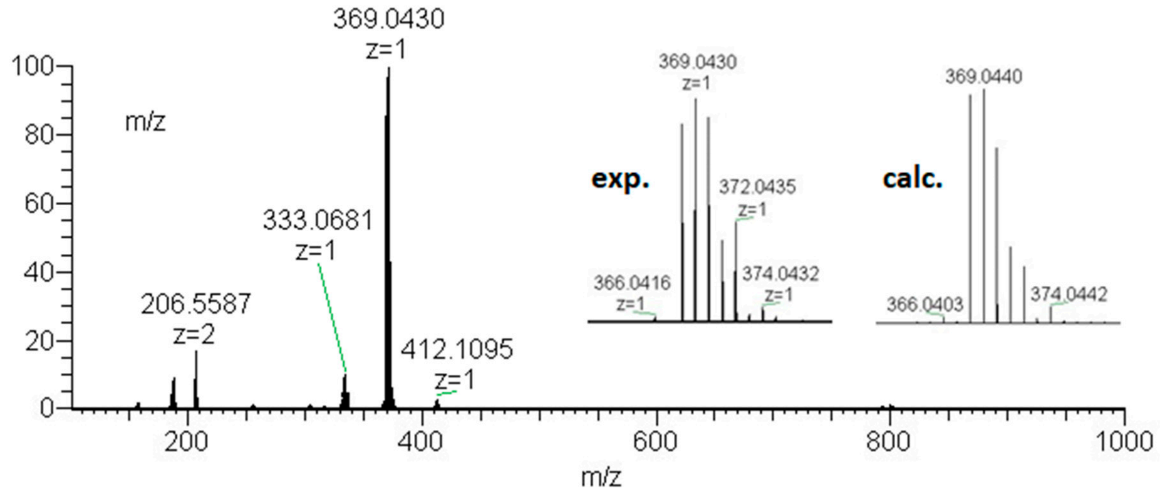

Figure S1: HR-ESI-MS spectrum of the complex [Pt(en)(py)Cl]NO_3 (**1**) in H_2O at 298K.

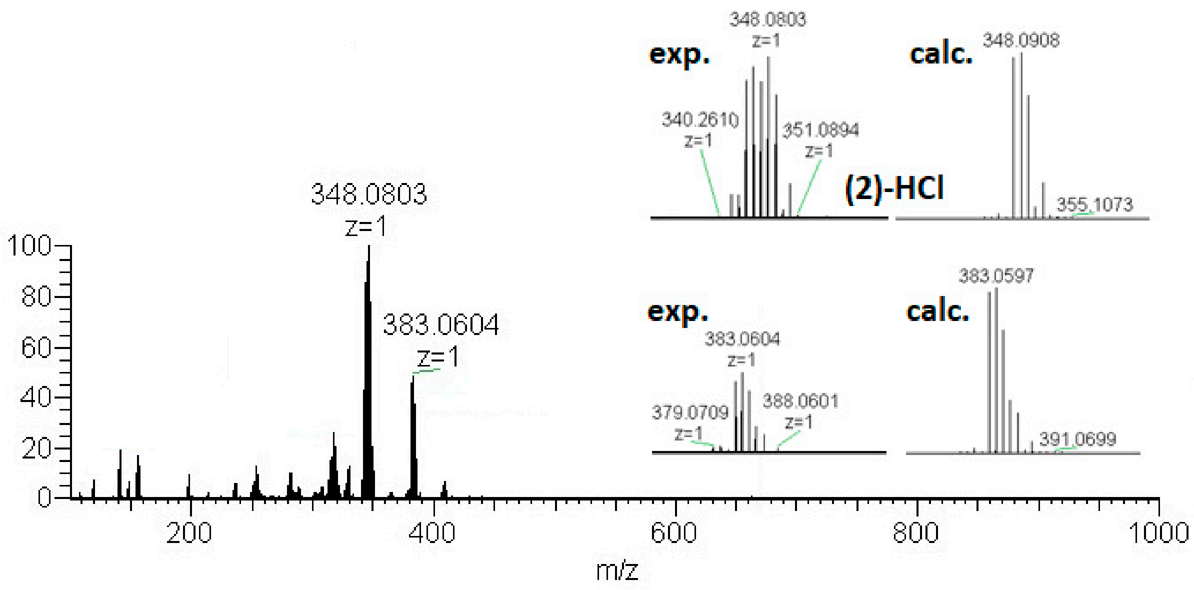

Figure S2: HR-ESI-MS spectrum of the complex [Pt(en)(2-mepy)Cl]NO_3 (**2**) in H_2O at 298K.

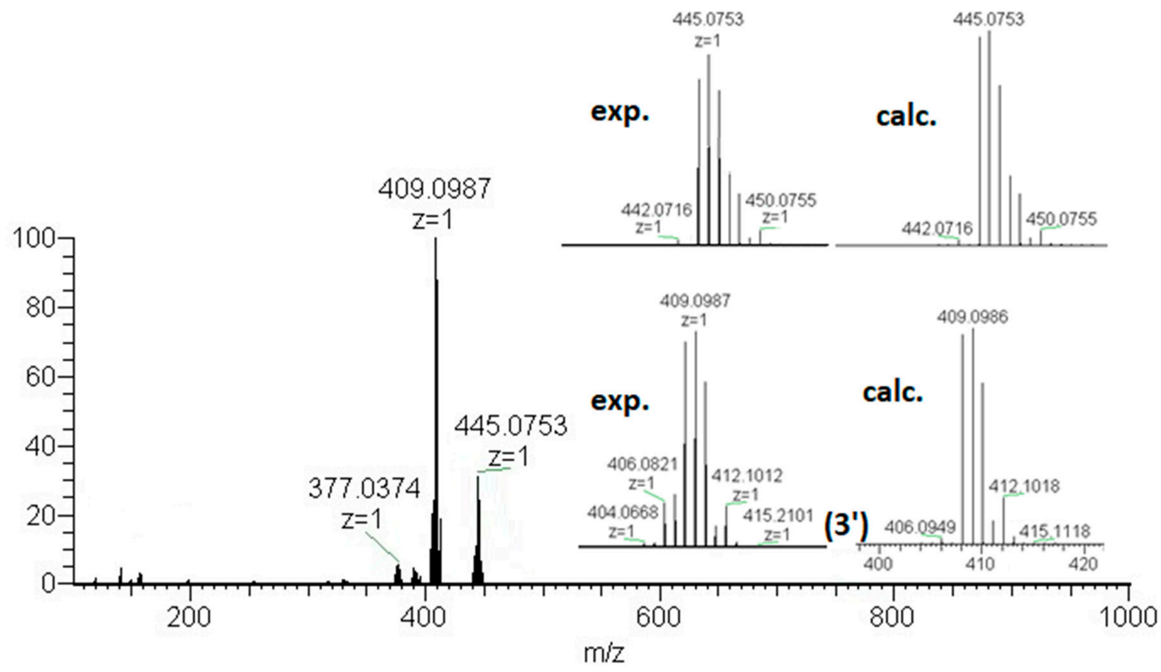

Figure S3: HR-ESI-MS spectrum of the complex [Pt(en)(2-phpy)Cl]Cl (**3**) and [Pt(en)(2-phpy)]Cl (**3'**) in H_2O at 298K.

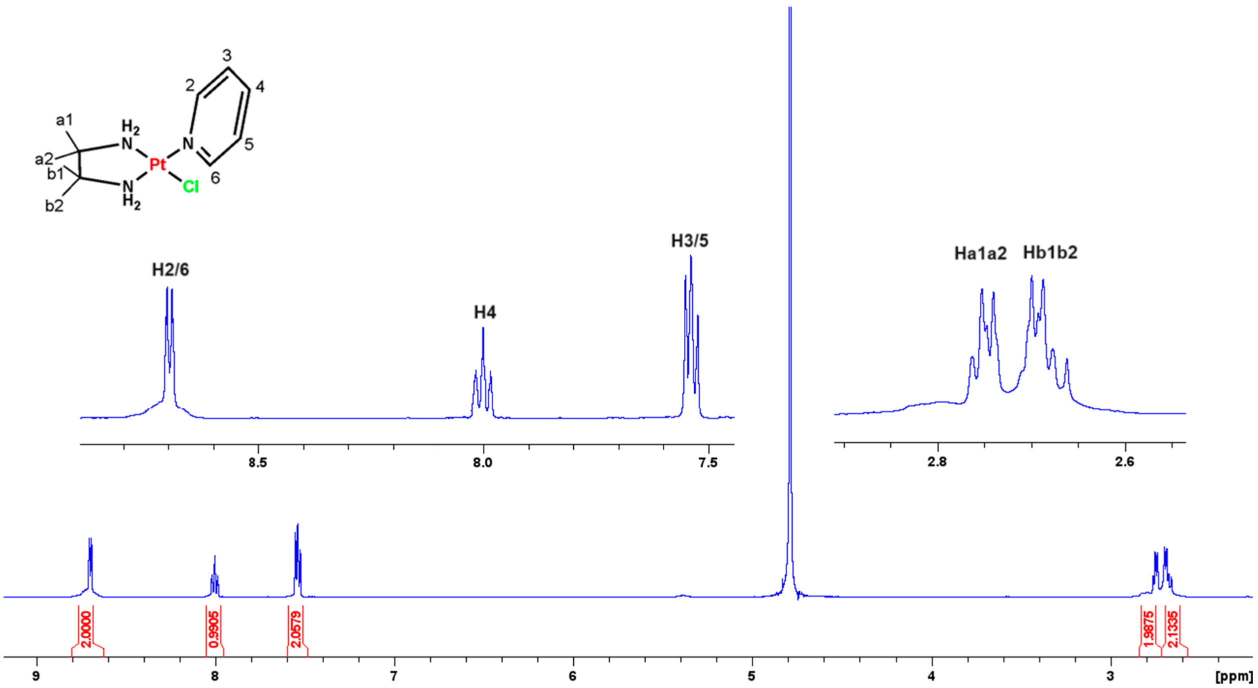

Figure S4: ^1H NMR of the complex [Pt(en)(py)Cl]NO_3 (**1**) inD_2O at 298K.

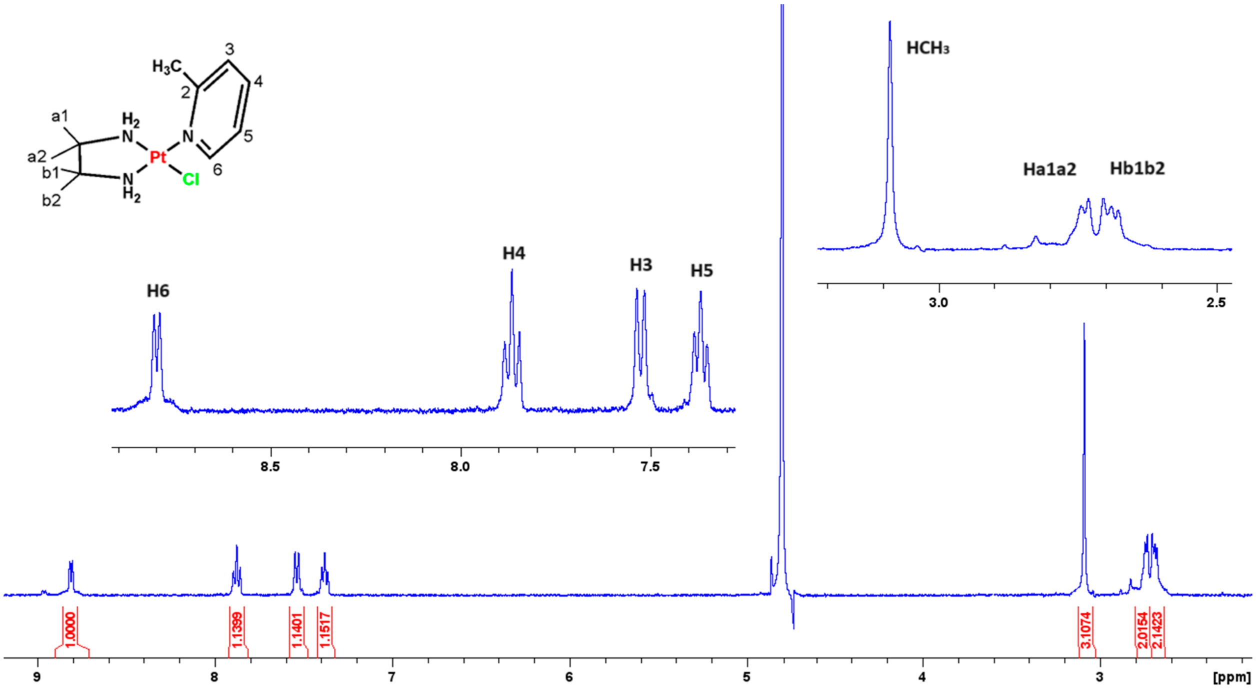

Figure S5: ^{1}H NMR of the complex [Pt(en)(2-mepy)Cl]NO_3 (**2**) in D_2O at 298K.

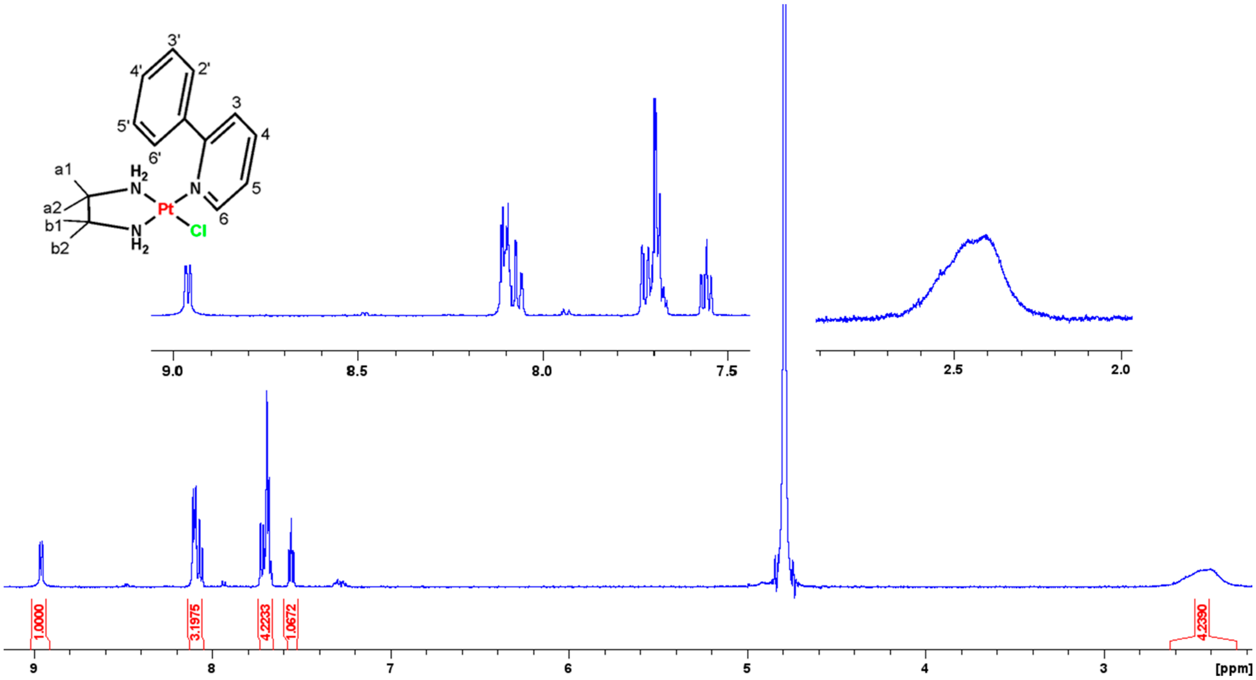

Figure S6: ^{1}H NMR of the complex [Pt(en)(2-phpy)Cl]Cl (**3**) in D_2O at 298K.

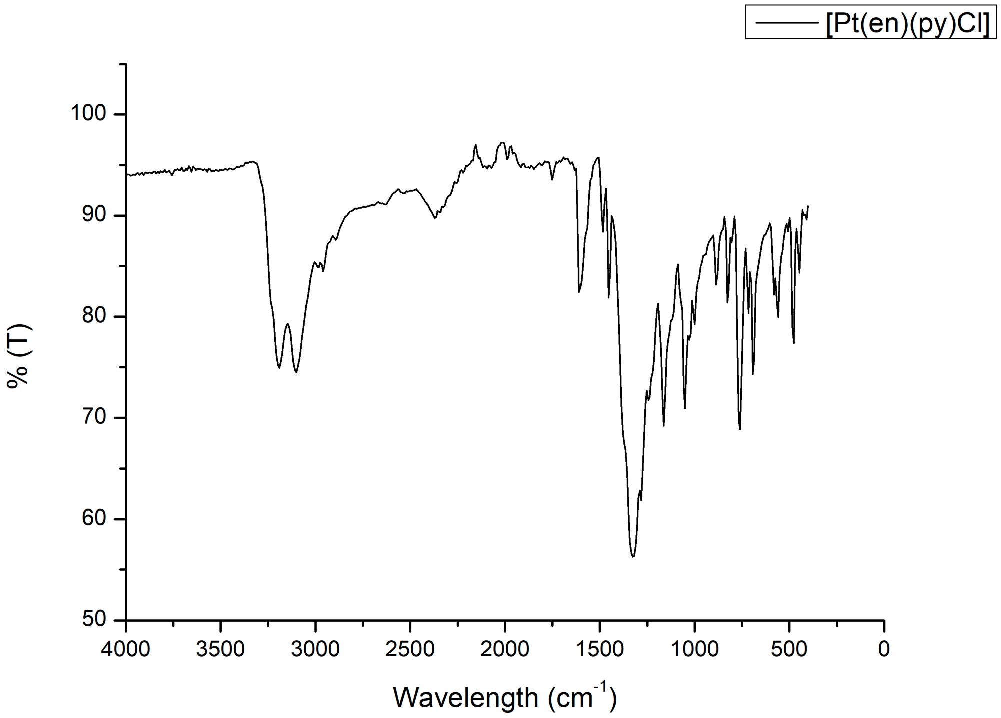

Figure S7: IR spectrum of [Pt(en)(py)Cl]NO_3 (**1**) at 298 K.

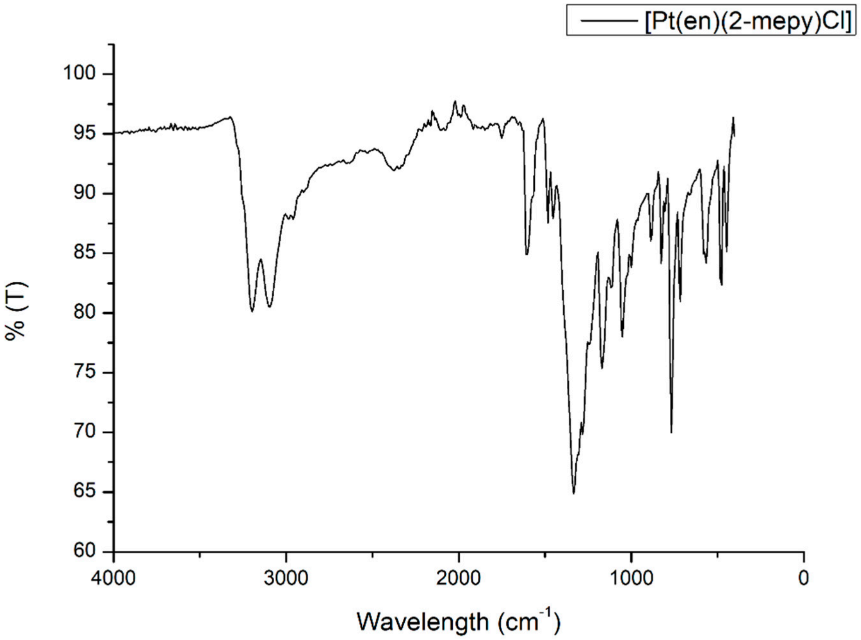

Figure S8: IR spectrum of [Pt(en)(2-mepy)Cl]NO_3 (**2**) at 298K.

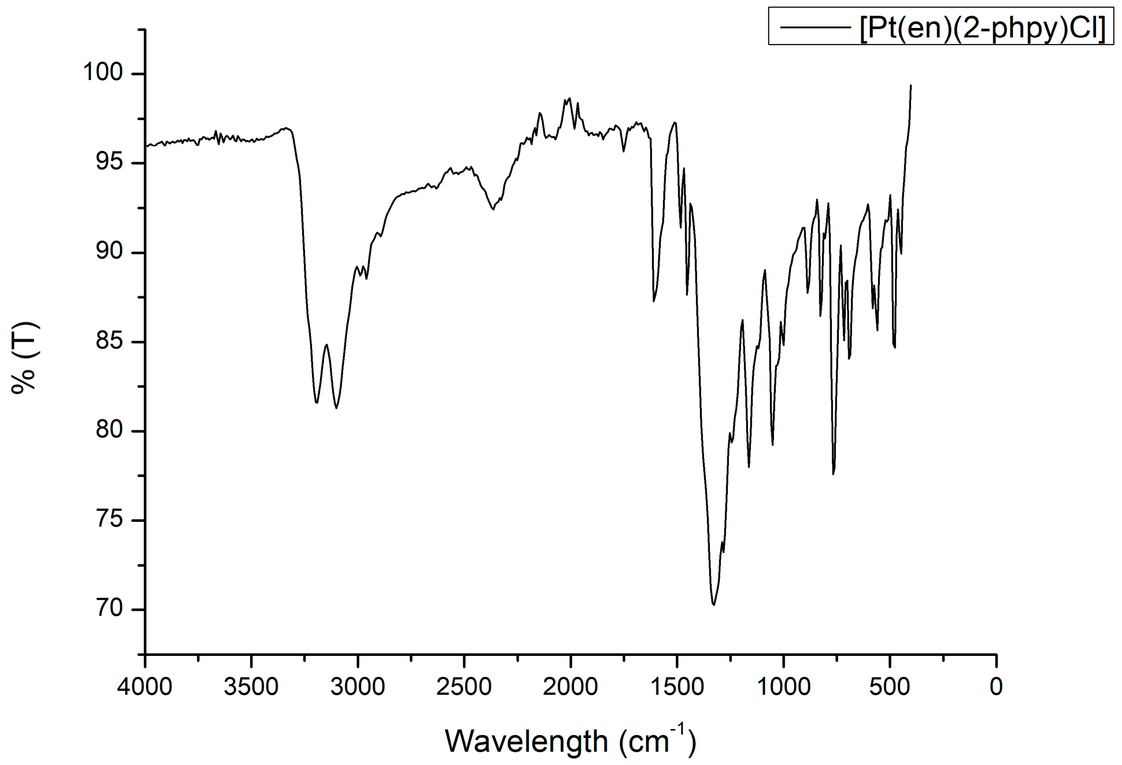

Figure S9: IR spectrum of [Pt(en)(2-phpy)Cl]Cl (**3**) at 298K.

Table S1: Concentrations of the hydrolyzed complex (**1**), [**1**-H_2O], as functions of time (t).

Time (x 10^2 sec)	[1-H_2O] (mM)
0	0
9	0.0621
18	0.124
27	0.210
36	0.255
72	0.370
108	0.443
252	0.454
864	0.462
1728	0.454

Table S2: Concentrations of the hydrolyzed complex (**2**), [**2**-H_2O], as functions of time (t).

**Time (x 10^2 sec)**	**[2-H_2O] (mM)**
0	0
9	0.0771
18	0.0805
27	0.0849
36	0.109
72	0.185
144	0.326
252	0.392
864	0.427
1728	0.417

Table S3: ^{13}C NMR data for selected species.

	**guanosine**						**pyridine ligand**						**en**	
	C8	C1′	C2′	C3′	C4′	C5′	C2	C3	C4	C5	C6	-CH_3	Ca	Cb
guo	138.6	88.1	73.9	70.9	85.9	61.8								
[**1**-D_2O]	-	-	-	-	-	-	152.6	127.8	141.1	127.8	152.6		47.9	47.9
[**1**-guo]	139.9	89.3	74.2	70.2	85.9	61.1	152.7	127.6	141.0	127.6	152.7		47.9	47.9
[**2**-guo]A	139.9	89.4	74.2	70.1	85.8	61.1	-	127.9	140.6	124.5	152.9	30.1	48.0	48.0
[**2**-guo]B	139.7	89.4	74.3	70.2	85.8	61.1	-	127.9	140.6	124.5	152.9	30.1	48.0	48.0

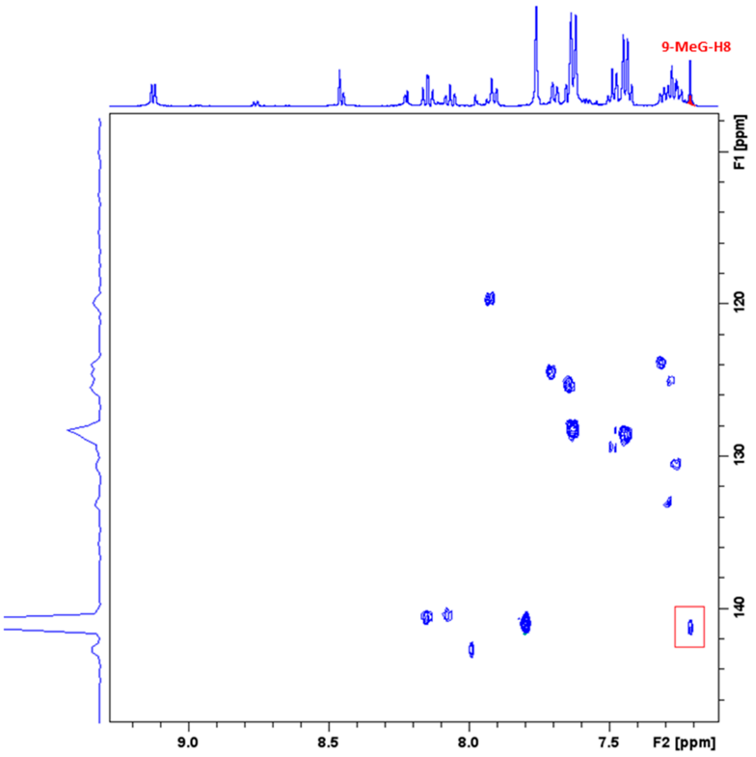

Figure S10: HSQC of the mixture of complex (**3**) and 9-methylguanine with NaCl 5 mM at 310K.
